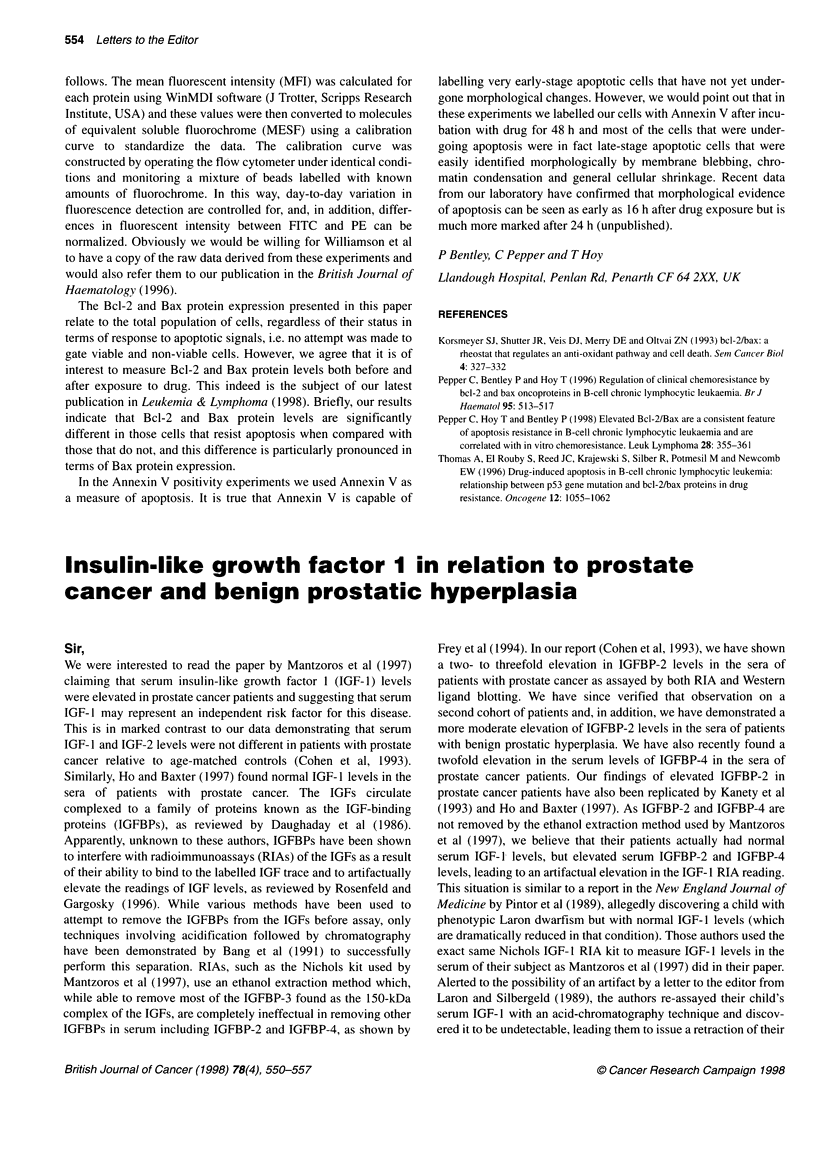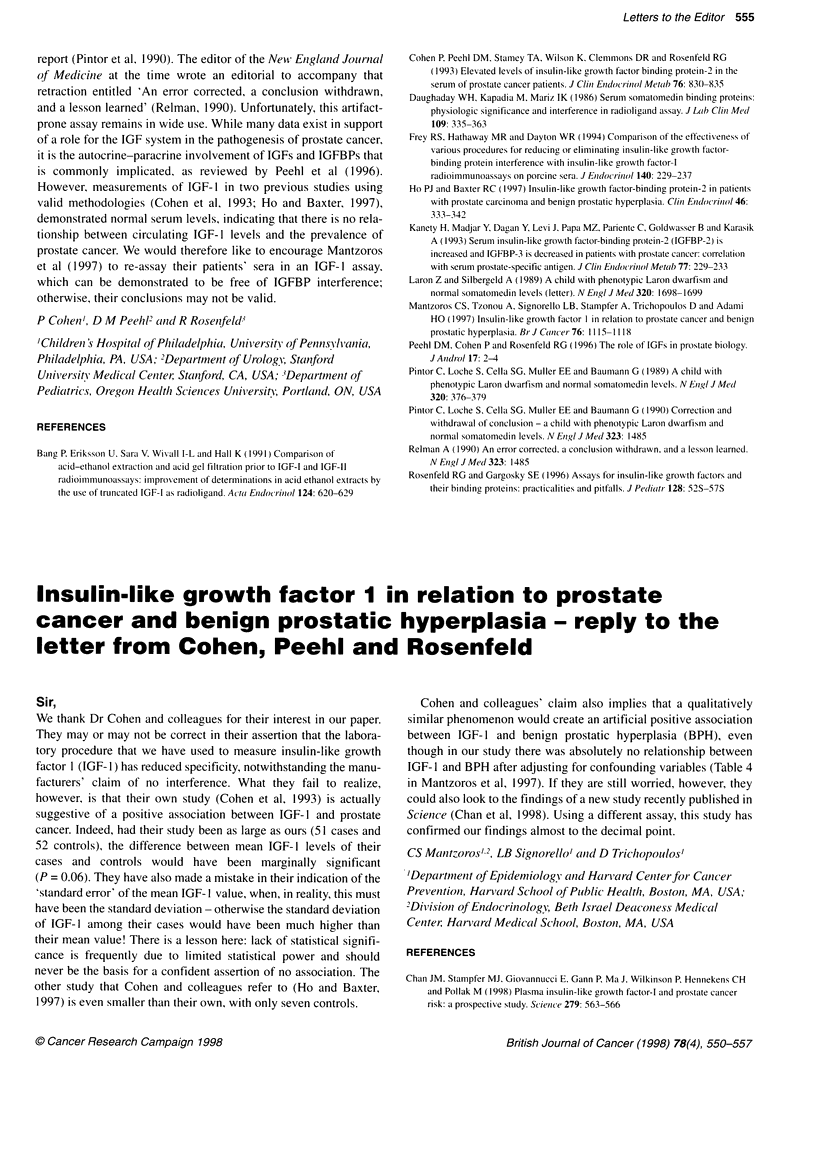# Insulin-like growth factor 1 in relation to prostate cancer and benign prostatic hyperplasia.

**DOI:** 10.1038/bjc.1998.535

**Published:** 1998-08

**Authors:** P. Cohen, D. M. Peehl, R. Rosenfeld


### Insulin-like growth factor 1 in relation to prostate cancer and benign prostatic hyperplasia

**Sir,**

We were interested to read the paper by Mantzoros et al (1997) claiming that serum insulin-like growth factor 1 (IGF-1) levels were elevated in prostate cancer patients and suggesting that serum IGF-1 may represent an independent risk factor for this disease. This is in marked contrast to our data demonstrating that serum IGF-1 and IGF-2 levels were not different in patients with prostate cancer relative to age-matched controls (Cohen et al, 1993). Similarly, Ho and Baxter (1997) found normal IGF-1 levels in the sera of patients with prostate cancer. The IGFs circulate complexed to a family of proteins known as the IGF-binding proteins (IGFBPs), as reviewed by Daughaday et al (1986). Apparently, unknown to these authors, IGFBPs have been shown to interfere with radioimmunoassays (RIAs) of the IGFs as a result of their ability to bind to the labelled IGF trace and to artifactually elevate the readings of IGF levels, as reviewed by Rosenfeld and Gargosky (1996). While various methods have been used to attempt to remove the IGFBPs from the IGFs before assay, only techniques involving acidification followed by chromatography have been demonstrated by Bang et al (1991) to successfully perform this separation. RIAs, such as the Nichols kit used by Mantzoros et al (1997), use an ethanol extraction method which, while able to remove most of the IGFBP-3 found as the 150-kDa complex of the IGFs, are completely ineffectual in removing other IGFBPs in serum including IGFBP-2 and IGFBP-4, as shown by Frey et al (1994). In our report (Cohen et al, 1993), we have shown a two- to threefold elevation in IGFBP-2 levels in the sera of patients with prostate cancer as assayed by both RIA and Western ligand blotting. We have since verified that observation on a second cohort of patients and, in addition, we have demonstrated a more moderate elevation of IGFBP-2 levels in the sera of patients with benign prostatic hyperplasia. We have also recently found a twofold elevation in the serum levels of IGFBP-4 in the sera of prostate cancer patients. Our findings of elevated IGFBP-2 in prostate cancer patients have also been replicated by Kanety et al (1993) and Ho and Baxter (1997). As IGFBP-2 and IGFBP-4 are not removed by the ethanol extraction method used by Mantzoros et al (1997), we believe that their patients actually had normal serum IGF-1 levels, but elevated serum IGFBP-2 and IGFBP-4 levels, leading to an artifactual elevation in the IGF-1 RIA reading. This situation is similar to a report in the *New England Journal of Medicine* by Pintor et al (1989), allegedly discovering a child with phenotypic Laron dwarfism but with normal IGF-1 levels (which are dramatically reduced in that condition). Those authors used the exact same Nichols IGF-1 RIA kit to measure IGF-1 levels in the serum of their subject as Mantzoros et al (1997) did in their paper. Alerted to the possibility of an artifact by a letter to the editor from Laron and Silbergeld (1989), the authors re-assayed their child's serum IGF-1 with an acid-chromatography technique and discovered it to be undetectable, leading them to issue a retraction of their

© Cancer Research Campaign 1998

report (Pintor et al, 1990). The editor of the *New England Journal of Medicine* at the time wrote an editorial to accompany that retraction entitled 'An error corrected, a conclusion withdrawn, and a lesson learned' (Relman, 1990). Unfortunately, this artifact-prone assay remains in wide use. While many data exist in support of a role for the IGF system in the pathogenesis of prostate cancer, it is the autocrine–paracrine involvement of IGFs and IGFBPs that is commonly implicated, as reviewed by Peehl et al (1996). However, measurements of IGF-1 in two previous studies using valid methodologies (Cohen et al, 1993; Ho and Baxter, 1997), demonstrated normal serum levels, indicating that there is no relationship between circulating IGF-1 levels and the prevalence of prostate cancer. We would therefore like to encourage Mantzoros et al (1997) to re-assay their patients' sera in an IGF-1 assay, which can be demonstrated to be free of IGFBP interference; otherwise, their conclusions may not be valid.

*P Cohen[1], D M Peehl[2] and R Rosenfeld[3]*

*[1]Children's Hospital of Philadelphia, University of Pennsylvania, Philadelphia, PA, USA; [2]Department of Urology, Stanford University Medical Center, Stanford, CA, USA; [3]Department of Pediatrics, Oregon Health Sciences University, Portland, ON, USA*

### REFERENCES

Bang P, Eriksson U, Sara V, Wivall I-L and Hall K (1991) Comparison of acid–ethanol extraction and acid gel filtration prior to IGF-I and IGF-II radioimmunoassays: improvement of determinations in acid ethanol extracts by the use of truncated IGF-I as radioligand. *Acta Endocrinol* **124**: 620–629

Cohen P, Peehl DM, Stamey TA, Wilson K, Clemmons DR and Rosenfeld RG (1993) Elevated levels of insulin-like growth factor binding protein-2 in the serum of prostate cancer patients. *J Clin Endocrinol Metab* **76**: 830–835

Daughaday WH, Kapadia M, Mariz IK (1986) Serum somatomedin binding proteins: physiologic significance and interference in radioligand assay. *J Lab Clin Med* **109**: 335–363

Frey RS, Hathaway MR and Dayton WR (1994) Comparison of the effectiveness of various procedures for reducing or eliminating insulin-like growth factor-binding protein interference with insulin-like growth factor-I radioimmunoassays on porcine sera. *J Endocrinol* **140**: 229–237

Ho PJ and Baxter RC (1997) Insulin-like growth factor-binding protein-2 in patients with prostate carcinoma and benign prostatic hyperplasia. *Clin Endocrinol* **46**: 333–342

Kanety H, Madjar Y, Dagan Y, Levi J, Papa MZ, Pariente C, Goldwasser B and Karasik A (1993) Serum insulin-like growth factor-binding protein-2 (IGFBP-2) is increased and IGFBP-3 is decreased in patients with prostate cancer: correlation with serum prostate-specific antigen. *J Clin Endocrinol Metab* **77**: 229–233

Laron Z and Silbergeld A (1989) A child with phenotypic Laron dwarfism and normal somatomedin levels (letter). *N Engl J Med* **320**: 1698–1699

Mantzoros CS, Tzonou A, Signorello LB, Stampfer A, Trichopoulos D and Adami HO (1997) Insulin-like growth factor 1 in relation to prostate cancer and benign prostatic hyperplasia. *Br J Cancer* **76**: 1115–1118

Peehl DM, Cohen P and Rosenfeld RG (1996) The role of IGFs in prostate biology. *J Androl* **17**: 2–4

Pintor C, Loche S, Cella SG, Muller EE and Baumann G (1989) A child with phenotypic Laron dwarfism and normal somatomedin levels. *N Engl J Med* **320**: 376–379

Pintor C, Loche S, Cella SG, Muller EE and Baumann G (1990) Correction and withdrawal of conclusion – a child with phenotypic Laron dwarfism and normal somatomedin levels. *N Engl J Med* **323**: 1485

Relman A (1990) An error corrected, a conclusion withdrawn, and a lesson learned. *N Engl J Med* **323**: 1485

Rosenfeld RG and Gargosky SE (1996) Assays for insulin-like growth factors and their binding proteins: practicalities and pitfalls. *J Pediatr* **128**: 52S–57S